# Gender-Based Violence Risk Mitigation by Non-GBV Specialists Prior to and during COVID-19: A Global Survey of Knowledge, Attitudes and Practices of Humanitarian Practitioners

**DOI:** 10.3390/ijerph182413387

**Published:** 2021-12-20

**Authors:** Vandana Sharma, Annika Gompers, Jocelyn T. D. Kelly, Erin Patrick, Christine Heckman, Arsema Solomon, Jennifer Scott

**Affiliations:** 1 Department of Global Health and Population, Harvard T.H. Chan School of Public Health, Boston, MA 02115, USA; 2 Harvard Humanitarian Initiative, Cambridge, MA 02138, USA; jtdkelly@gmail.com; 3 Department of Obstetrics and Gynecology, Beth Israel Deaconess Medical Center, Boston, MA 02215, USA; agompers@bidmc.harvard.edu (A.G.); arsemasol@gmail.com (A.S.); jscott3@bidmc.harvard.edu (J.S.); 4 Department of Emergency Medicine, Brigham and Women’s Hospital, Boston, MA 02115, USA; 5 CARE, New York, NY 10017, USA; Erin.Patrick@care.org; 6 United Nations Children’s Fund, New York, NY 10038, USA; checkman@unicef.org; 7 Department of Obstetrics, Gynecology, and Reproductive Biology, Harvard Medical School, Boston, MA 02115, USA

**Keywords:** gender-based violence, humanitarian contexts, COVID-19, GBV risk mitigation, humanitarian response, protection, humanitarian aid

## Abstract

Available evidence indicates that the COVID-19 pandemic and response measures may lead to increased risk of gender-based violence (GBV), including in humanitarian contexts. This study examined the knowledge, attitudes, and practices of humanitarian practitioners related to GBV risk mitigation approaches during COVID-19 in order to refine current guidance and inform future materials. A global, online cross-sectional survey of humanitarian practitioners was conducted between November 2020 and April 2021. We calculated descriptive statistics and used Chi-square or Fisher’s exact tests to compare knowledge, attitudes, and practices among GBV specialists and non-specialists. Of 170 respondents, 58% were female and 44% were GBV specialists. Almost all (95%) of the respondents agreed or strongly agreed that they have a role to play in GBV risk mitigation. Compared to GBV specialists, a higher proportion of non-specialists reported little to no knowledge on GBV risk mitigation global guidance (38% vs. 7%, *p* < 0.001) and on how to respond to a disclosure of GBV (18% vs. 3%, *p* < 0.001). Respondents reported several barriers to integrating GBV risk mitigation into their work during COVID-19, including insufficient funding, capacity, knowledge, and guidance. Efforts to mainstream GBV risk mitigation actions should continue and intensify, leveraging the lessons and experiences generated thus far.

## 1. Background

Available evidence demonstrates that the risk of gender-based violence (GBV) increases during humanitarian crises [1,2]. Although GBV is prevalent in all contexts, emergencies disrupt protection structures and create circumstances that can lead to even greater risks of violence. For example, risks of GBV during crises are often exacerbated by factors such as separation from family members, disruption of social support systems and social cohesion, shifting responsibilities of family members, poverty, emotional stress, and lack of safe access to basic needs [3,4]. Sequelae of GBV include: poor physical and mental health outcomes [5,6,7], unintended pregnancy, and increased vulnerability to sexually transmitted infections, including HIV [8,9,10]. Survivors may also have difficulty seeking healthcare, be at greater risk for substance abuse and face stigma and negative social outcomes in their families and communities [11,12].

Recent reports suggest that efforts to contain the spread of COVID-19, such as quarantine and movement restrictions, may lead to increased risk of GBV, including intimate partner violence (IPV) [13,14]. These measures aim to control transmission of the novel coronavirus (SARS-CoV-2), but—in the case of IPV—they also limit survivors’ abilities to distance themselves from perpetrators, while simultaneously reducing their access to GBV response services, such as clinical care, and mental health/psychosocial support. In addition, there has been widespread job loss, economic strain, and stress and other mental health outcomes due to the pandemic that likely contribute to the increased risk of GBV [15,16].

Given the far-reaching economic effects of the pandemic, there is an increased risk of sexual exploitation and abuse (SEA), as households are more likely to face shortages of resources or become dependent on humanitarian assistance [17]. This is not a new issue. For example, prior to the pandemic, several high-profile examples of SEA involving aid workers demanding sex in exchange for food, supplies or other forms of humanitarian aid have been documented [18,19,20]. There may also be increased checkpoints and posted security personnel as part of efforts to enforce COVID-19 restrictions, which could elevate the risk of various forms of GBV, as was the case during the Ebola epidemic [21,22]. Women and girls may also face heightened exposure to GBV risk at COVID-19 quarantine centers, where inadequate lighting, limited presence of qualified staff, and overcrowding could increase risk of violence. School closures may also increase risk of GBV, as girls who are not in school could be exposed to increased violence at home and have less access to existing referral pathways and other forms of support available through the education system [23,24].

Addressing GBV during emergencies includes three pillars of action: (1) implementing **prevention** efforts that target the root causes of violence and seek to prevent GBV from occurring in the first place; (2) providing specialized **response** services for survivors of violence, addressing the consequences of GBV after it has happened; and (3) **risk mitigation** actions which aim to reduce exposure to GBV and ensure that humanitarian response actions and services themselves do not cause harm or increase risk of violence (See Table 1) [25,26].

Gender-based violence risk mitigation actions are a collective responsibility across the entire humanitarian system, as articulated by the principles of Centrality of Protection and Do No Harm. As such, they should be systematically integrated in the design, implementation, and evaluation of actions to respond to disasters and emergencies, across all sectors, and by all humanitarian practitioners. The foundational guidance document on GBV risk mitigation for all humanitarian sectors and actors is the InterAgency Standing Committee (IASC)’s 2015 *Guidelines for Integrating GBV Interventions in Humanitarian Action* (“GBV Guidelines”), a revised version of the first set of guidelines, originally launched in 2005 [25]. The IASC is the longest-standing and highest-level coordination forum of the UN system comprising 18 heads of UN and non-UN organizations. The GBV Guidelines include specialized guidance for the following sectors: Camp Coordination and Camp Management (CCCM), Child Protection, Education, Food Security and Agriculture, Health, Housing, Land and Property, Humanitarian Mine Action, Humanitarian Operations Support Sectors, Livelihoods, Nutrition, Protection, Shelter, and Water, Sanitation and Hygiene (WASH).

The GBV Guidelines distinguish between ‘GBV Specialists’ and ‘non-GBV specialists’, describing GBV specialists as practitioners with professional training in GBV and/or considerable experience working on GBV programming. The GBV Guidelines are primarily targeted towards non-GBV specialists who work in other non-GBV sectors and who do not have specific expertise in GBV programming, but who could undertake activities in the context of their work to reduce GBV risks for affected populations. The GBV Guidelines describe one role of GBV specialists as assisting non-GBV specialists to undertake GBV risk mitigation efforts in their own sectors and to serve as a technical resource. UNICEF leads the inter-agency efforts on GBV risk mitigation including revision and development of the GBV Guidelines, inter-agency implementation of GBV risk mitigation across the humanitarian system and funding a dedicated inter-agency coordinator for the GBV Guidelines Reference Group, consisting of 15 humanitarian agencies. The GBV Guidelines Implementation Support team has made progress in increasing knowledge, awareness, and uptake of the GBV Guidelines through trainings and other dissemination efforts, though some anecdotal evidence suggests varying levels of uptake by sector.

In addition to the GBV Guidelines, a step-by-step guide for humanitarian practitioners entitled *How to support survivors of gender-based violence when a GBV actor is not available in your area* (“GBV Pocket Guide”) was developed jointly by the GBV Guidelines Reference Group and the GBV Area of Responsibility (AoR), to help colleagues working in other (non-GBV) sectors to safely and appropriately respond to disclosures of GBV in humanitarian contexts [26].

While the existing humanitarian guidance and good practices are relevant in the current context, COVID-19 brings new challenges [27]. Given the additional risks and constraints created by the pandemic, some GBV risk mitigation strategies may require adaptation in order to be effective. For example, consultations with women and girls to better understand their risks and barriers to accessing services may need to be conducted through other modalities when there are limitations on in-person gatherings. Gender-based violence risk mitigation guidance tailored to COVID-19 is now available on behalf of the GBV Guidelines Reference Group through a complementary tip sheet called *Identifying & Mitigating Gender-based Violence Risks within the COVID-19 Response* [28]. It identifies key, sector-specific GBV risks that are likely to occur and/or be exacerbated during the COVID-19 response, along with recommendations on how to mitigate these risks. This guidance document was developed at the beginning of the pandemic, based on established good practice, but there is a need for more learning and evidence on GBV risk mitigation actions that have been implemented and/or adapted during the pandemic by non-GBV specialists in different sectors in order refine the current guidance and to inform future tools, materials, and training materials.

In order to fill these gaps, and inform future mainstreaming efforts, this research study was carried out to examine how humanitarian practitioners are incorporating GBV risk mitigation actions across all programmatic sectors during COVID-19, their knowledge, attitudes and practices around mitigation approaches and the barriers to integrating these interventions within their day-to-day work. Given that the important role of GBV specialists in providing technical support and serving as a key resource for non-GBV specialists in carrying out GBV risk mitigation actions, we sought to compare knowledge, attitudes and practices and perceived barriers to implementation across these two groups.

## 2. Methods

### 2.1. Study Design

This is a cross-sectional study nested within the Humanitarian Gender Study, a larger mixed-methods study whose overall aim is to understand gender bias in the humanitarian sector including during active emergencies such as COVID-19. The quantitative component of the Humanitarian Gender Study included a gender bias survey and a GBV risk mitigation and COVID-19 survey. The GBV risk mitigation and COVID-19 survey aimed to better understand humanitarian practitioners’ knowledge, attitudes, and experience in mitigating GBV risks within the context of COVID-19, as well as how humanitarian programming during the pandemic has been adapted and is affecting GBV risks. This survey was conducted between November 2020 and April 2021 by collaborators at the Beth Israel Deaconess Medical Center (BIDMC), Harvard T.H. Chan School of Public Health, Harvard Humanitarian Initiative, Brigham and Women’s Hospital, UNICEF and CARE. The cross-sectional design allowed for rapid deployment and wide-spread dissemination to reach potential respondents around the world through social media platforms and humanitarian networks. The qualitative methods and findings that were part of the Humanitarian Gender Study will be described elsewhere.

### 2.2. Setting

We implemented this study online and globally through REDCap—a secure, HIPAA compliant, web-based data collection tool hosted at BIDMC [29]. The survey was available in English, French, Spanish, Arabic, Amharic, and Swahili, which reflects some of the major languages employed by humanitarian practitioners in humanitarian crises across the world.

### 2.3. Participant Recruitment, Sampling and Sample Size

The survey was disseminated primarily through listservs and online networks, including organizations, research groups, professional networks, and social media groups (Facebook and LinkedIn) focused on humanitarian practitioners. Messages sent through these networks included a link to a consent form and the online survey and to the study’s website (www.humanitariangenderstudy.org, accessed on 18 October 2021). We also published Facebook and LinkedIn advertisements targeted towards employees of major humanitarian organizations. These advertisements linked to the same REDCap consent and survey page and website. Finally, direct outreach was conducted by study team members to humanitarian practitioners within our own networks to raise awareness about the study and to enable further sharing and dissemination. The inclusion criteria included self-identification as age 18 years and older, ability to complete the survey in one of the six available languages, and current or past paid or unpaid work related to humanitarian assistance. Work could be in any role including administrative, programming, research, and policy. Humanitarian assistance was defined as assistance that is intended to save lives, alleviate suffering, and maintain human dignity after man made crises and disasters caused by natural hazards, as well as to prevent and strengthen preparedness for when such situations occur. Respondents were excluded from the sample if they did not meet all of the inclusion criteria or if they had not completed at least one of the non-demographic survey modules.

### 2.4. Questionnaire and Measures

The questionnaire was developed in English by a team with experience in public health, humanitarian assistance, and GBV risk mitigation and was piloted among a small group of humanitarian practitioners. The questionnaire was translated to the five additional languages and the translations were independently reviewed and corrected. The survey included questions about participants’ demographics; employment information and how employment status and location may have been affected by COVID-19; experience with GBV response and prevention; general knowledge, attitudes and practices related to GBV risk mitigation; local COVID-19 response measures and their perceived impacts on GBV risk; integration and effectiveness of GBV risk mitigation intervention within sector-specific programming pre-pandemic and during COVID-19; programmatic adaptations to mitigate GBV risk during COVID-19; and knowledge and use of GBV guidelines. As we were particularly interested in understanding GBV risk mitigation in non-protection sectors and among practitioners who do not directly work on GBV, we collected self-reported information on the humanitarian sectors that the respondent’s work falls under and whether or not they identify as a GBV specialist. A GBV specialist was defined as a humanitarian professional with specialized GBV knowledge and expertise (such as GBV program managers or Interagency GBV Coordinators) regardless of whether they work in protection or non-protection sectors.

### 2.5. Statistical Analysis

We analyzed responses collected between 17 November 2020 and 14 April 2021. We received a total of 541 completed quantitative surveys as part of the Humanitarian Gender Study: 371 responses to the gender bias survey and 170 responses to the GBV risk mitigation and COVID-19 survey. Data analysis focused on generating descriptive statistics which are reported as a frequency (percentage). We used Chi-square or Fisher’s exact tests to compare GBV risk mitigation attitudes, experience, and practices among individuals self-describing as GBV specialists and those who reported as non-specialists, regardless of which sector their work was conducted in. Analyses were performed in SAS 9.4 (SAS Institute, Cary, NC, USA).

### 2.6. Ethical Review

All of the participants provided informed consent to engage in this research after reviewing a detailed Information Sheet presented in English, Amharic, Arabic, French, Spanish or Swahili at the beginning of the REDCap survey. The study was conducted in accordance with the Declaration of Helsinki, and the protocol was approved by the Institutional Review Board of BIDMC (Protocol 2020P000618). All research staff associated with this study completed CITI Program’s Research, Ethics and Compliance Training.

### 2.7. Role of the Funding Source

The funders of the study had no role in study design, data collection, data analysis, data interpretation, or writing of the report. The corresponding author had full access to all of the data in the study and had final responsibility for the decision to submit for publication.

## 3. Results

### 3.1. Respondent Characteristics

The sample included 170 humanitarian practitioners (58% female) who completed at least one of the survey modules (Table 2). The majority of the responses were submitted in English (76%). Participants reported working in 52 different countries, with 7% reporting that they work globally. The majority of practitioners reported working for International NGOs (51%), followed by National NGOs (24%), UN and related organizations (18%), as well as a range of other types of organizations such as Community-based Organizations (CBOs), local women’s organizations, donors, research institutions and governments. Approximately 52% of respondents reported that their work relates to more than one humanitarian sector. Overall, 52% of respondents reported that their work involves the Protection/GBV sector, followed by Health (36%), Child Protection (26%), Education (21%), Livelihoods (19%), and WASH (15%). Approximately 44% of respondents reported that they are GBV specialists. The average percentage of time spent in the field prior to COVID-19 was 41%, compared to 28% during the pandemic. In addition, 7% of respondents identified as living with a disability, 11.2% identified as being a displaced person, refugee, or asylum seeker, and 5% identified as part of the LGBTQ+ community.

### 3.2. Knowledge, Attitudes and Practices Related to GBV Risk Mitigation

The majority of respondents in the sample (95%) and across sectors agreed or strongly agreed that as humanitarian professionals, they have a role to play in GBV risk mitigation (Table 3 & Appendix A). However, there were variations in the proportion of respondents who agreed or strongly agreed that GBV risk mitigation does not fall within the scope their own work, from 10% in the Protection sector, to 27% in Shelter, and 29% in the Education sector (Appendix A). Overall, 27% of respondents agreed that sector-specific work is a priority over addressing GBV, though again there was variation by sector. In addition, 59% of practitioners agreed with the statement that they would like to work on GBV risk mitigation but there are limited financial resources, while 28% agreed that they do not have the knowledge or skills, 20% agreed that they do not have the support of their supervisor to work on GBV risk mitigation, and 14% agreed that they do not have the time.

Approximately 24% of the sample reported little to no knowledge on GBV risk mitigation global guidance and this proportion was significantly higher among non-GBV specialists compared to GBV specialists (Table 3, 38% vs. 7%, *p* < 0.001). Similarly, a significantly higher proportion of non-GBV specialists reported little to no knowledge on how to respond to a disclosure of GBV compared to GBV specialists (18% vs. 3%, *p* < 0.001). GBV risk mitigation experience was also significantly higher among GBV specialists. Over 86% of GBV specialists reported having implemented GBV risk mitigation activities at least once during a humanitarian emergency, compared with 50% of non-GBV specialists (*p* < 0.001).

### 3.3. Humanitarian Programs and Perceptions of GBV Risk during COVID-19

Across the sample, 89% of humanitarian practitioners reported that humanitarian programming had been greatly or moderately affected by the COVID-19 pandemic, and 65% reported that availability and access to GBV services were greatly or moderately reduced due to the pandemic (Table 4). Changes in GBV risk were also reported by the majority of respondents. Almost 80% of respondents reported that GBV risks greatly or moderately increased due to the COVID-19 pandemic and associated response measures. A very small number of respondents reported that GBV risk had decreased (2%), and several respondents reported that they did not know whether risks had changed. The forms of GBV for which risk was perceived to have increased include IPV (85%), emotional/psychological violence (79%), socioeconomic violence (71%), sexual exploitation/transactional sex (61%), child and/or forced marriage (53%), sexual harassment (52%), rape and non-partner sexual violence (44%) and female genital cutting (13%). These data demonstrate perceived risks as reported by the survey respondents and should not be confused with incidence or prevalence data which were not collected as part of this study. When asked their perceptions about which groups faced greater risk, respondents stated these groups were: adolescent girls (87%), adult women (85%), people with disabilities (52%), elderly women (37%), adolescent boys (26%), individuals of non-conforming sexual /gender identities (26%), and adult men (17%). There was greatest consensus among the sample around increased GBV risks for adolescent girls and adult women.

### 3.4. Perceptions of GBV Risk Mitigation Integration, Adaptations, and Effectiveness

Non-GBV specialized sectors can integrate GBV risk mitigation into their work by taking specific actions to identify and understand GBV risks associated with their programming, implementing, or adapting interventions to address those risks, and measuring outcomes regarding access or safety perceptions of those adaptations. When asked about the degree of integration of GBV risk mitigation interventions within their main sector of focus, 65% respondents reported great or moderate level of integration prior to the COVID-19 pandemic, compared with 67% during the pandemic (Table 5). There was no statistical difference in the level of reported integration between GBV and non-GBV specialists. The majority of practitioners reported that GBV risk mitigation interventions had been adapted during COVID-19 (61%) (Table 6). The most common types of adaptations included changing the modality or implementation of consultations with women and girls (71%), changing the modality of service delivery (65%), including GBV information in COVID-19 education materials (65%), changing the timing or frequency of service delivery (58%), providing up-to-date information about referral pathways (54%), and increasing coordination with GBV specialists (45%). Very few respondents reported stopping service delivery (9%) as a means of mitigating GBV risk during the COVID-19 pandemic. There were no statistical differences in reported adaptations between non-GBV and GBV specialists.

Specific examples of adaptations included leveraging technology to conduct virtual assessments, telephone-based safety planning, as well as use of WhatsApp and social media for dissemination of GBV messaging. However, lack of access to mobile phones and technology was noted as a challenge for these approaches, especially for women and girls. Other creative approaches to disseminating GBV information such as through leaflets in food baskets and through loudspeakers/megaphones and radio programming were also noted. A number of examples of increasing cash assistance, including through mobile distribution, to alleviate financial stress were shared, although it was also noted that increasing the amount of cash disbursed may inadvertently increase the risk of GBV.

Overall, only 31% of practitioners who reported integrating GBV risk mitigation interventions in their sector-specific work, reported that these interventions were very effective or fairly effective (Table 6). Almost 55% reported that these interventions were somewhat effective, slightly effective, or not effective at all, while close to 15% reported that they were unsure of the interventions’ effectiveness. Almost twice as many GBV specialists as non-GBV specialists reported these interventions as very or fairly effective, a difference that was statistically significant (41.9% vs. 21.9%, *p* = 0.02). The most common reasons provided for the limited effectiveness of the GBV risk mitigation interventions include not enough funding (62.4%), COVID-19 restrictions (52.7%), prioritization of other issues (46.2%), insufficient staff capacity or knowledge (43%), not enough guidance or gaps in the guidance (41.9%), lack of organizational commitment to addressing GBV risk (38.7%), insufficient human resources (38.7%), and barriers to accessing guidance (26.9%). The proportion of humanitarians reporting these barriers was generally similar between GBV and non-GBV specialists. However, 52% of non-GBV specialists cited lack of GBV risk mitigation guidance as a barrier compared to only 25.7% of GBV specialists (*p* = 0.01).

## 4. Discussion

This study finds that while humanitarian practitioners in the sample almost unanimously agree that all humanitarian workers have a role to play in GBV risk mitigation (in alignment with the IASC GBV Guidelines), there are reported gaps in knowledge and experience, and barriers to integration of these actions within day-to-day work during COVID-19. For example, almost 40% of non-GBV specialists in this study reported little or no knowledge of the GBV Guidelines. This signifies that about 60% of interviewed non-GBV specialists do have some knowledge of this foundational guidance document, which is an encouraging sign about efforts to raise awareness. However, almost 45% of the interviewed non-GBV specialists reported insufficient knowledge or skills to carry out GBV risk mitigation within their usual sector-specific work. Even among those who report knowledge and skills, several types of perceived barriers to putting GBV risk mitigation into practice were described.

The limited knowledge or skills on GBV risk mitigation reported by practitioners in our study, especially among non-GBV actors, aligns with research conducted before the pandemic. For instance, a series of case studies focused on Central African Republic, South Sudan, Iraq and Sierra Leone found limited understanding of how to operationalize the IASC GBV Guidelines, and lack of consistency in the way that donors, UN agencies and other implementing partners interpreted, prioritized and implemented the guidelines [30]. Gender-based violence was often considered “too multifaceted or complex for concrete emergency response programming.” [30]. However, these case studies were developed to assess the 2005 IASC GBV Guidelines and were published shortly after the launch of the revised guidelines and rollout of trainings. There is a paucity of published data on knowledge and use of the revised 2015 GBV Guidelines. Our study sheds some light on these topics and suggests that, at least among the practitioners in our sample, similar challenges are occurring, especially among the non-protection actors.

For example, practitioners in our study also reported lack of organizational commitment to GBV risk mitigation and prioritization of other areas as barriers to the implementation of GBV risk mitigation interventions. Several factors may underly low commitment and prioritization. For example, lack of GBV expertise at the field level and within senior management within organizations inhibits the prioritization of GBV interventions within humanitarian response plans [31]. Lack of prioritization may also be linked to patriarchal culture in decision-making structures, and socio-cultural perceptions, biases and attitudes towards gendered programming [32]. Other studies build on this by suggesting that far more effort is needed to address gender inequality within the broader humanitarian system including by supporting female leadership and activism and by increasing the number of women in leadership positions [33]. Addressing these issues requires tackling underlying gender biases within humanitarian organizations [34].

Both GBV and non-GBV specialists in our study reported that lack of funding has been a key barrier to integrating effective GBV risk mitigation within their work both before and during COVID-19. This finding is particularly interesting for several reasons. First, higher level commitments to addressing GBV have been visible during the COVID-19 pandemic, including a joint statement by 146 UN member states and observers supporting prioritization of measures to address GBV in national COVID-19 response plans [35]. Our findings suggest that public declarations by governments and decision-makers may not have translated into tangible actions such as resource allocation or increased availability of funds at ground-level for GBV risk mitigation. This might be at least partially explained by the fact that many of the public statements and commitments to address GBV have focused on GBV response for survivors and have mainly targeted the health sector. Gender-based violence risk mitigation has not been well addressed within these commitments. Second, there is some existing evidence of inadequate funding for GBV within the humanitarian sector, at least for GBV programs, gathered before the pandemic. For example, an IRC and VOICE study found that GBV program funding accounted for only 0.12% of all humanitarian funding between 2016 and 2018; but this report did not examine GBV risk mitigation [31]. In general, there are limited data on funding for GBV risk mitigation within the humanitarian sector. Third, and most importantly, it is interesting that practitioners in this study highlight lack of funding as a major barrier, when many GBV risk mitigation interventions do not require additional funds. This perspective could be a reflection of the limited knowledge on GBV risk mitigation interventions within the sample. Further awareness raising on the range of GBV risk mitigation interventions that are feasible, and on the resources required may be helpful. On the other hand, this attitude could potentially also be a way to justify inaction, where lack of funding is given as an excuse for not taking the time to analyze what is possible with little to no funds. Importantly, the programming components that are required to ensure safe and accessible programming are integral to the programming itself, and budget lines for these risk mitigation components must be included in funding requests.

The same VOICE and IRC study that assessed funding gaps also highlighted that localization of humanitarian action, including related to GBV, has remained low [31]. Localization refers to increasing the power and access of local actors to decision-making and funding [36]. This approach leads to a faster, more effective, and more sustainable humanitarian response because local actors better understand the complex social and political environment and have greater access to affected populations [37]. Localization is important for effective GBV prevention, response and mitigation in emergencies, as well [33], since local women’s organizations have context-specific expertise and deep understanding of women’s and girls’ specific risks and needs. Given their expertise, engaging these organizations could also help address some of the staff capacity and knowledge gaps that were raised by practitioners in our study. Yet, there also remain significant funding gaps for women-focused organizations during COVID-19, who, for example, have been left out of humanitarian response funding in Asia [38,39]. 

Humanitarian practitioners in this study also shared their perceptions of the changing GBV risk environment during COVID-19 within the contexts in which they work. Most practitioners in this study reported the possibility of increases in risk of IPV during the pandemic; this aligns with growing reports and published research [40]. Additionally, survey respondents reported perceptions that risks of other forms of violence, including SEA, sexual harassment and child or forced marriage, also increased during the pandemic. Respondents also reported that the pandemic could result in elevated risks of emotional/psychological violence and socioeconomic violence, which often receive limited focus. These findings correspond with other reports. For instance, a recent qualitative study in Ethiopia found that the COVID-19 pandemic has increased the risk of child marriage for adolescent girls and some adolescent boys due to school closures, lack of surveillance and interruption of community monitoring mechanisms of possible cases of child marriage by teachers and other informants [41]. A study in Nigeria reported police misconduct including cases of sexual harassment and sexual assault by police enforcing COVID-19 lockdown measures [42].

Our study sheds important light on the different types of programmatic adaptations that are being made to address GBV risks in emergencies during the pandemic. Notably, changing the modality of consultations with women and girls and of service delivery were the most common adaptations reported, followed closely by incorporating GBV information within COVID-19 education materials. These are similar to examples of adaptations included in other reports [30]. Singh et al. provide examples of using digital technology and social media to reach target populations and incorporating GBV information within other programming such as inclusion of GBV hotline numbers on cash aid program cards in Haiti [43]. Our study builds on these examples to provide a more comprehensive view on the types of adaptations that are occurring in humanitarian contexts across the world, with a particular eye towards GBV risk mitigation in non-GBV sectors. It is important to note that existing reports do not fully examine potential risks associated with these adaptations and how these are being mitigated on the ground. For instance, shifting to remote or technology-based approaches may help ensure that services are not interrupted in the face of COVID-19 restrictions, but they may introduce new risks such as online harassment. Furthermore, many settings have limited internet connectivity and access to mobile devices, and often access is most limited among women and girls. Abusive partners may also restrict women’s access to different forms of technology. Some of these challenges have been reported in our global survey and strategies to address these issues will be further explored in the study’s qualitative data, which will be analyzed separately.

## 5. Strengths and Limitations

This study has several limitations. First, the use of social media and humanitarian networks to recruit study subjects restricts the generalizability of the findings. Several countries, such as Ethiopia, are over-represented in the sample because of the study teams’ larger networks in this setting. Respondents recruited through social media or humanitarian networks may differ from others humanitarian professionals in several ways including literacy, access to technology, and resources. The survey was available in six languages, but practitioners fluent in other languages may not have been able to participate. Data were collected across countries that were at different stages of the pandemic during the study period. This may have limited the examples of GBV risk mitigation adaptations collected as settings with fewer restrictions may not have required adapted approaches. Despite intense efforts to recruit respondents, the sample size for this component of the study was lower than anticipated, and this reduced power to conduct comparisons across the 13 humanitarian sectors. The low sample size was possibly due to the highly specialized topic of the survey and survey fatigue, particularly given the significant increase in online data collection processes that accompanied the COVID-19 response. Despite these limitations, the study has several strengths. To our knowledge, this is the first study to systematically assess GBV risk mitigation in COVID-19 with a focus on non-GBV specialists among a global sample of humanitarian practitioners. The study was able to capture perspectives across 52 countries and 13 humanitarian clusters, demonstrating a wide reach across numerous humanitarian contexts and types of emergencies. The instrument developed for this research was designed to robustly capture a wide range of indicators on GBV risk mitigation including knowledge, attitudes, and practices, among both GBV and non-GBV specialists and will help to strengthen and advance measurement of GBV risk mitigation, an area that has significant gaps [44].

## 6. Conclusions

In summary, the study findings suggest that despite increasing efforts to mainstream GBV risk mitigation across the humanitarian system, there are reported knowledge and skills gaps, especially among non-GBV specialists and across sectors, and barriers to implementing GBV risk mitigation interventions both prior to and during the COVID-19 pandemic. Our study provides unique contributions to the expanding field of GBV risk mitigation and highlights the need for the following actions.

- Efforts to mainstream GBV risk mitigation actions, in particular strengthening knowledge and skills-building, addressing unsupportive attitudes, and overcoming barriers to implementation and effectiveness should not only continue during the COVID-19 pandemic, but should accelerate while leveraging the lessons and experience generated thus far. Complementary to this work, strategies to increase prioritization of GBV risk mitigation in non-GBV sectors should continue to be explored and tested across humanitarian organizations. This may require addressing gender biases and other factors that influence decision-making, promoting women’s leadership in the humanitarian sector and generating evidence to demonstrate how addressing specific GBV risks could also improve sector-specific outcomes [33];
- Misconceptions on GBV risk mitigation, such as the belief that additional and/or separate funding is always required to carry out this work, should be addressed. Additional awareness raising about the range of GBV risk mitigation intervention options, including those that can be implemented with little to no additional funding, would be helpful. For those risk mitigation interventions which do require additional funds, sectors have a responsibility to include budget for GBV risk mitigation within their own funding requests to ensure their programming is safe and accessible during COVID-19 and beyond. Currently across the humanitarian sector, there is no standard way of calculating or tracking GBV risk mitigation expenditure within sectors’ total budgets. Systematizing this type of tracking would be valuable for the field, as it would facilitate understanding and identification of funding gaps and ensure that available resources match the need;
- Awareness and knowledge of global guidance on GBV risk mitigation was limited among non-GBV specialists in this study, though the stated desire for guidance was high among this group. Therefore, intensifying efforts to promote uptake and use of the available guidance targeting those practitioner groups with less access could help bridge the gap. Donors could also support these efforts by referencing the IASC GBV Guidelines—and/or requesting specific content be included (GBV risk analysis, indicators, etc.)—within project proposals;
- The range and scope of adaptations to GBV risk mitigation efforts during the COVID-19 pandemic has demonstrated how organizations and practitioners have creatively pivoted during an uncertain, challenging and highly dynamic situation. Further efforts to document the rich learning and experiences of practitioners engaged in this work would be beneficial;
- As reduced availability and access to GBV services has been reported in conjunction with perceived increases in GBV-related risks due to the COVID-19 pandemic and associated restrictions, ramping up of GBV risk mitigation interventions is more crucial than ever. These efforts must be prioritized and implemented in tandem with GBV prevention and response efforts. In addition, resources such as the GBV Pocket Guide [26] should be leveraged and scaled up;
- Further research and evaluations are needed to more robustly assess the effectiveness of specific GBV risk mitigation interventions, both within and outside of the COVID-19 response, and to better understand what works and what does not in different sectors. This will ensure that practitioners who are already overstretched can concentrate their efforts along with the limited available resources on those interventions that will have the greatest impact.

## Figures and Tables

**Table 1:** The three pillars of GBV action in humanitarian emergencies. Adapted from the IASC GBV Guidelines training materials [25].

	GBV Prevention	GBV Risk Mitigation	GBV Response
**What**	Interventions to prevent GBV from first occurring	Interventions that reduce exposure to GBV and ensure that humanitarian response actions and services themselves do not cause harm or increase risk of violence	Interventions to address the consequences of GBV after it has happened
**How**	Address root causes of violence such as gender inequalities and social norms	Proactively identify and, to the extent possible, address contributing factors	Provide specialized response services to survivors of GBV
**Who**	Could be carried out by GBV specialists, but also other humanitarian actors if appropriate	Responsibility of all humanitarian actors, governments, communities, everyone	GBV, Health and Protection Specialists who have appropriate training
**Examples**	Community awareness and social norms interventions to promote positive gender norms and equal power dynamics	Reduce exposure to GBV by addressing overcrowding, improving safety at access points (food, water, health services), adequate lighting, appropriate shelter division, gender-segregated latrines	Case management, mental health and psychosocial support, clinical care, legal support

**Table 2:** Demographic and employment characteristics of humanitarian practitioners in the study sample.

	All N = 170	GBV Specialists N = 74	Non-GBV Specialists N = 96
	N (%)	N (%)	N (%)
**Survey Language**			
English	129 (75.9)	56 (75.7)	73 (76.0)
French	17 (10.0)	8 (10.8)	9 (9.4)
Arabic	13 (7.7)	5 (6.8)	8 (8.3)
Spanish	11 (6.5)	5 (6.8)	6 (6.3)
**Gender Identity**			
Female	98 (57.7)	49 (66.2)	49 (51.0)
Male	69 (40.6)	24 (32.4)	45 (46.9)
Prefer not to say or Other	3 (1.8)	1 (1.4)	2 (2.1)
**Country where the majority of respondent’s work with affected populations takes place (52 total)**			
Ethiopia	25 (14.7)	12 (16.2)	13 (13.5)
Global	11 (6.5)	7 (9.5)	4 (4.2)
Uganda	11 (6.5)	2 (2.7)	9 (9.4)
Colombia	9 (5.3)	3 (4.1)	6 (6.3)
Mali	9 (5.3)	3 (4.1)	6 (6.3)
Syria	9 (5.3)	5 (6.8)	4 (4.2)
Kenya	8 (4.7)	6 (8.1)	2 (2.1)
A different country	88 (51.8)	36 (48.6)	52 (54.2)
**Living in a setting affected by a humanitarian emergency**			
Yes	47 (27.7)	21 (28.4)	26 (27.1)
**Displaced person/refugee/asylum seeker**			
Yes	19 (11.2)	9 (12.2)	10 (10.4)
**Living with a disability**			
Yes	12 (7.1)	6 (8.1)	6 (6.3)
**LGBTQI+**			
Yes	8 (4.7)	3 (4.1)	5 (5.2)
**Education Completed**			
High school or equivalent	4 (2.4)	2 (2.7)	2 (2.1)
Bachelor’s degree	40 (23.5)	12 (16.2)	28 (29.2)
Master’s degree	89 (52.4)	43 (58.1)	46 (47.9)
Professional degree	10 (5.9)	4 (5.4)	6 (6.3)
Doctorate	12 (7.1)	7 (9.5)	5 (5.2)
Technical/trade	1 (0.6)	0 (0.0)	1 (1.0)
Other or missing	3 (1.8)	6 (8.1)	8 (8.3)
Employment status *			
Employed full time	142 (83.5)	64 (86.5)	78 (81.3)
Employed part time	17 (10.0)	7 (9.5)	10 (10.4)
Unemployed	5 (2.9)	2 (2.7)	3 (3.1)
In education or training	8 (4.7)	3 (4.1)	5 (5.2)
Retired	1 (0.6)	1 (1.4)	0 (0.0)
Other	2 (1.2)	0 (0.0)	2 (2.1)
**Years in the humanitarian sector**			
<1 year	3 (1.8)	2 (2.7)	1 (1.0)
1–4.9 years	50 (29.4)	21 (28.4)	29 (30.2)
5–9.9 years	52 (30.6)	20 (27.0)	32 (33.3)
10–14.9 years	33 (19.4)	13 (17.6)	20 (20.8)
15 or more years	32 (18.8)	18 (24.3)	14 (14.6)
Cluster *			
Camp Coordination and Camp Management	12 (7.1)	4 (5.4)	8 (8.3)
Child Protection	44 (25.9)	18 (24.3)	26 (27.1)
Education	35 (20.6)	12 (16.2)	23 (24.0)
Food Security & Agriculture	22 (12.9)	8 (10.8)	14 (14.6)
Health	61 (35.9)	23 (31.1)	38 (39.6)
Housing, Land, and Property	7 (4.1)	3 (4.1)	4 (4.2)
Humanitarian Mine Action	2 (1.2)	2 (2.7)	0 (0.0)
Humanitarian Operations Support Sectors	21 (12.4)	5 (6.8)	16 (16.7)
Livelihoods	33 (19.4)	10 (13.5)	23 (24.0)
Nutrition	20 (11.8)	5 (6.8)	15 (15.6)
Protection/GBV	89 (52.4)	53 (71.6)	36 (37.5)
Shelter	11 (6.5)	3 (4.1)	8 (8.3)
WASH	25 (14.7)	5 (6.8)	20 (20.8)
Other	32 (18.8)	10 (13.5)	22 (22.9)
Type of emergency/population *			
Active conflict	54 (31.8)	31 (41.9)	23 (24.0)
Disaster/natural hazard	42 (24.7)	19 (25.7)	23 (24.0)
Stable protracted emergency	55 (32.4)	26 (35.1)	29 (30.2)
Post-conflict/post-disaster	61 (35.9)	30 (40.5)	31 (32.3)
Urban	67 (39.4)	32 (43.2)	35 (36.5)
Rural	61 (35.9)	27 (36.5)	34 (35.4)
Displacement camp/settlement	54 (31.8)	31 (41.9)	23 (24.0)
IDPs	76 (44.7)	38 (51.4)	38 (39.6)
Refugees	78 (45.9)	37 (50.0)	41 (42.7)
Host population(s)	70 (41.2)	37 (50.0)	33 (34.4)
Other	16 (9.4)	7 (9.5)	9 (9.4)
**GBV specialist**			
Yes	74 (43.5)	74 (100.0)	0 (0.0)
Type of organization *			
UN and related organizations	30 (17.7)	16 (21.6)	14 (14.6)
International NGO	86 (50.6)	40 (54.1)	46 (47.9)
National NGO	40 (23.5)	18 (24.3)	22 (22.9)
Community Based Organization (CBO)	12 (7.1)	6 (8.1)	6 (6.3)
Local women’s organization	9 (5.3)	8 (10.8)	1 (1.0)
Academic/research	12 (7.1)	3 (4.1)	9 (9.4)
Government	9 (5.3)	3 (4.1)	6 (6.3)
Private sector	9 (5.3)	1 (1.4)	8 (8.3)
Health facility	3 (1.8)	1 (1.4)	2 (2.1)
Consultancy firm	2 (1.2)	1 (1.4)	1 (1.0)
Self-employed	8 (4.7)	6 (8.1)	2 (2.1)
Donor	2 (1.2)	0 (0.0)	2 (2.1)
Faith-based organization	5 (2.9)	2 (2.7)	3 (3.1)
Military	1 (0.6)	1 (1.4)	0 (0.0)
Other	6 (3.5)	1 (1.4)	5 (5.2)
Current roles and responsibilities *			
Human resources	26 (15.3)	12 (16.2)	14 (14.6)
Professional development	68 (40.0)	35 (47.3)	33 (34.4)
Program administration/management	100 (58.8)	47 (63.5)	53 (55.2)
M&E	69 (40.6)	29 (39.2)	40 (41.7)
Management of field-based work	34 (20.0)	17 (23.0)	17 (17.7)
Organizational/institutional policies	47 (27.7)	27 (36.5)	20 (20.8)
Engagement with beneficiaries	71 (41.8)	35 (47.3)	36 (37.5)
Media/communications/Public relations	32 (18.8)	12 (16.2)	20 (20.8)
Advocacy	52 (30.6)	30 (40.5)	22 (22.9)
Other	14 (8.2)	5 (6.8)	9 (9.4)
**Percentage of time in the field—before COVID-19**			
	40.9 ± 29.9	38.8 ± 25.1	42.5 ± 33.2
**Percentage of time in the field—during COVID-19**			
	28.1 ± 29.7	23.6 ± 21.4	31.7 ± 34.9

* Respondent could select multiple options.

**Table 3:** GBV Risk Mitigation Knowledge, Attitudes and Practices of GBV and non-GBV specialists.

	All (N = 170)	GBV Specialists (N = 74)	Non-GBV Specialists (N = 96)	*p*-Value *
	N (%)	N (%)	N (%)	
**Attitudes towards GBV RM—those who agree/strongly agree ^†^**				
As a humanitarian professional, I have a role to play in GBV risk mitigation	162 (95.3)	72 (97.3)	90 (93.8)	0.47
Only GBV specialists should work to mitigate risks of GBV	12 (7.1)	6 (8.1)	6 (6.3)	0.64
It is not within my scope of work to mitigate risks of GBV	22 (12.9)	5 (6.8)	17 (17.7)	0.03
Sector-specific work is a priority over addressing GBV	45 (26.5)	19 (25.7)	26 (27.1)	0.84
I would like to work to mitigate risks of GBV, but I do not have the support of my supervisor(s)/senior management to do so	34 (20.0)	12 (16.2)	22 (22.9)	0.28
I would like to work to mitigate risks of GBV, but there are limited financial resources	101 (59.4)	44 (59.5)	57 (59.4)	0.99
I would like to work to mitigate risks of GBV, but I do not have the time	23 (13.5)	5 (6.8)	18 (18.8)	0.02
I would like to work to mitigate risks of GBV, but I do not have the knowledge or skills	48 (28.2)	5 (6.8)	43 (44.8)	<0.001
**GBV RM knowledge—those with little or no knowledge ^‡^**				
Global guidance on GBV RM in humanitarian programming	41 (24.1)	5 (6.8)	36 (37.5)	<0.001
How to respond if a survivor discloses an experience of GBV and asks for your help	19 (11.2)	2 (2.7)	17 (17.7)	0.002
Measuring GBV RM outcomes in your sector-specific humanitarian programming	43 (25.3)	8 (10.8)	35 (36.5)	<0.001
Asking about safety perceptions of women and girls in your sector-specific humanitarian programming	33 (19.4)	1 (1.4)	32 (33.3)	<0.001
**GBV RM Experience & Practices**				
Have implemented GBV RM activities at least once in a humanitarian emergency	112 (65.9)	64 (86.5)	48 (50.0)	<0.001
Day-to-day work never or rarely involves GBV RM efforts	46 (27.1)	7 (9.5)	39 (40.6)	<0.001

* *p*-values calculated by Chi-square or Fisher’s exact test. ^†^ Answer options were: Strongly agree, Agree, Neutral, Disagree, and Strongly Disagree. ^‡^ Answer options were: Expert knowledge, A lot of knowledge, Some knowledge, A little knowledge, and No knowledge. GBV RM: GBV risk mitigation.

**Table 4:** Humanitarian programs & perceived GBV risk during COVID-19.

	All	GBV Specialists	Non-GBV Specialists	*p*-Value *
	N (%)	N (%)	N (%)	
**How much has humanitarian programming been affected by COVID-19**	**N = 170**	**N = 74**	**N = 96**	
To a great extent	93 (54.7)	42 (56.8)	51 (53.3)	0.18
To a moderate extent	58 (34.1)	28 (37.8)	30 (31.3)	
To a small extent	7 (4.1)	1 (1.3)	6 (6.3)	
Not at all	4 (2.4)	2 (2.7)	2 (2.1)	
I don’t know	8 (4.7)	1 (1.4)	7 (7.3)	
**Reduced availability/access to GBV services due to COVID-19 (N = 170)**	**N = 170**	**N = 74**	**N = 96**	
To a great extent	56 (32.9)	30 (40.5)	26 (27.1)	0.01
To a moderate extent	54 (31.8)	28 (37.8)	26 (27.1)	
To a small extent	30 (17.7)	10 (13.5)	20 (20.8)	
Not at all	7 (4.1)	3 (4.1)	4 (4.2)	
I don’t know	23 (13.5)	3 (4.1)	20 (20.8)	
**Change in GBV risk due to COVID-19**	**N = 170**	**N = 74**	**N = 96**	
Increased to a great extent	94 (55.3)	47 (63.5)	47 (49.0)	0.20
Increased to a moderate extent	41 (24.1)	19 (25.7)	22 (22.9)	
Increased to a small extent	9 (5.3)	3 (4.1)	6 (6.3)	
No change	4 (2.4)	1 (1.4)	3 (3.1)	
Decreased	4 (2.4)	1 (1.4)	3 (3.1)	
Some risks increased and some risks decreased	5 (2.9)	1 (1.4)	4 (4.2)	
I don’t know	13 (7.7)	2 (2.7)	11 (11.5)	
**Forms of GBV for which risk has increased ^‡^**	**N = 149**	**N = 70**	**N = 79**	
Intimate partner violence	126 (84.6)	58 (82.9)	68 (86.1)	0.59
Rape and non-partner sexual violence	65 (43.6)	36 (51.4)	29 (36.7)	0.07
Sexual exploitation / transactional sex	91 (61.1)	48 (68.6)	43 (54.4)	0.08
Early, child, and/or forced marriage	79 (53.0)	48 (68.6)	31 (39.2)	<0.001
Female genital cutting	20 (13.4)	15 (21.4)	5 (6.3)	0.01
Sexual harassment	78 (52.4)	39 (55.7)	39 (49.4)	0.44
Socioeconomic violence	105 (70.5)	48 (68.6)	57 (72.2)	0.63
Emotional/psychological violence	118 (79.2)	57 (81.4)	61 (77.2)	0.53
Other	4 (2.7)	3 (4.3)	1 (1.3)	0.34
I don’t know	3 (2.0)	0 (0.0)	3 (3.8)	0.25
**Groups for whom risk of GBV has increased ^‡^**	**N = 149**	**N = 70**	**N = 79**	
Adult women	126 (84.6)	63 (90.0)	63 (79.8)	0.08
Adult men	25 (16.8)	13 (18.6)	12 (15.2)	0.58
Adolescent girls	129 (86.6)	61 (87.1)	68 (86.1)	0.85
Adolescent boys	38 (25.5)	17 (24.3)	21 (26.6)	0.75
Elderly women	55 (36.9)	29 (41.4)	26 (32.9)	0.28
People with disabilities	78 (52.4)	42 (60.0)	36 (45.6)	0.08
Individuals of non-conforming sexual/gender identities	38 (25.5)	24 (34.3)	14 (17.7)	0.02
Other	3 (2.0)	2 (2.9)	1 (1.3)	0.6
I don’t know	9 (6.0)	4 (5.7)	5 (6.3)	1

* *p*-values calculated by Chi-square or Fisher’s exact test. **^‡^** Respondent could select multiple options.

**Table 5:** Integration of GBV risk mitigation before and during COVID-19.

	All (N = 144)	GBV Specialists (N = 70)	Non-GBV Specialists (N = 74)	*p*-Value
	N (%)	N (%)	N (%)	
**Integration of GBV risk mitigation in sector-specific work before COVID-19**				
To a small extent or not at all	50 (34.7)	22 (31.4)	28 (37.8)	0.42
To a great or moderate extent	94 (65.3)	48 (68.6)	46 (62.2)	
**Integration of GBV risk mitigation in sector-specific work during COVID-19**				
To a small extent or not at all	47 (32.6)	20 (28.6)	27 (36.5)	0.31
To a great or moderate extent	97 (67.4)	50 (71.4)	47 (63.5)	

**Table 6:** GV risk mitigation adaptations and effectiveness during COVID-19.

	All	GBV Specialists	Non-GBV Specialists	
	N (%)	N (%)	N (%)	*p*-Value *
**GBV RM adaptations during COVID-19**				
Among those integrating GBV RM, GBV RM strategies have been adapted during COVID-19 (N = 140)	104 (61.2)	56 (81.2)	48 (67.6)	0.07
Among those adapting GBV RM, adaptations have included (N = 104)				
Changing modality of service delivery	68 (65.4)	40 (71.4)	28 (58.3)	0.16
Changing timing/frequency of service delivery	60 (57.7)	36 (64.3)	24 (50.0)	0.14
Changing modality/implementation of consultations with women and girls	74 (71.2)	43 (76.8)	31 (64.6)	0.17
Stopping service delivery	9 (8.7)	5 (8.9)	4 (8.3)	1
Increased coordination/contact with GBV specialists	47 (45.2)	28 (50.0)	19 (39.6)	0.29
Providing up-to-date info about referral pathways	56 (53.9)	34 (60.7)	22 (45.8)	0.13
Setting up additional entry points to connect with GBV services	34 (32.7)	19 (33.9)	15 (31.3)	0.77
Including GBV information in COVID-19 education materials	68 (65.4)	37 (66.1)	31 (64.6)	0.87
Other	1 (1.0)	1 (1.8)	0 (0.0)	1
**Effectiveness of GBV RM during COVID-19**				
Level of effectiveness of GBV RM in your sector-specific work during COVID-19 (N = 170)				
Very or fairly effective	52 (30.6)	31 (41.9)	21 (21.9)	0.02
Somewhat effective or less	93 (54.7)	35 (47.3)	58 (60.4)	
Not sure/don’t know	25 (14.7)	8 (10.8)	17 (17.7)	
Reasons GBV risk mitigation not more effective * (N = 93)				
Not enough funding	58 (62.4)	23 (65.7)	35 (60.3)	0.6
Not enough guidance/gaps in guidance	39 (41.9)	9 (25.7)	30 (51.7)	0.01
Guidance not translated into my language	13 (14.0)	6 (17.1)	7 (12.1)	0.55
Barriers to access guidance (e.g., internet, ability to print)	25 (26.9)	8 (22.9)	17 (29.3)	0.5
Guidance not available in my preferred modality	8 (8.6)	1 (2.9)	7 (12.1)	0.25
Lack of organizational commitment to address GBV risk	36 (38.7)	11 (31.4)	25 (43.1)	0.26
Prioritizing other issues	43 (46.2)	19 (54.3)	24 (41.4)	0.23
COVID-19 restrictions	49 (52.7)	18 (51.4)	31 (53.5)	0.85
Insufficient human resources	36 (38.7)	12 (34.3)	24 (41.4)	0.5
Insufficient staff capacity/knowledge	40 (43.0)	16 (45.7)	24 (41.4)	0.68
Other	4 (4.3)	0 (0.0)	4 (6.9)	0.29

* *p*-values calculated by Chi-square or Fisher’s exact test, GBV RM: GBV risk mitigation.

## Data Availability

Survey materials are available by request to the corresponding author.

## Abbreviations

AoR	Area of Responsibility
BIDMC	Beth Israel Deaconess Medical Center
CBO	Community-based organization
FCDO	Foreign, Commonwealth & Development Office
GBV	Gender-based Violence
GBV RM	Gender-based Violence Risk Mitigation
SEA	Sexual Exploitation and Abuse
IASC	InterAgency Standing Committee
IPV	Intimate Partner Violence
NGO	Non-governmental organization
NIHR	National Institute for Health Research
R2HC	Research for Health in Humanitarian Crises
WASH	Water, Sanitation and Hygiene

## Appendix A. GBV Risk Mitigation Knowledge, Attitudes and Practices of Humanitarian Practitioners by Sector

**Table:**

		Protection Sectors		Non-Protection Sectors											
	All	Protection/GBV	Child Protection	CCCM	Education	Food Security & Agriculture	Health	Housing, Land, and Property	Humanitarian Mine Action	Humanitarian Operations Support Sectors	Livelihoods	Nutrition	Shelter	WASH	Other
	N = 170	N = 89	N = 44	N = 12	N = 35	N = 22	N = 61	N = 7	N = 2	N = 21	N = 33	N = 20	N = 11	N = 25	N = 32
	N (%)	N (%)	N (%)	N (%)	N (%)	N (%)	N (%)	N (%)	N (%)	N (%)	N (%)	N (%)	N (%)	N (%)	N (%)
**Attitudes towards GBV RM—those who agree/strongly agree**															
As a humanitarian professional, I have a role to play in GBV risk mitigation	162 (95.3)	88 (98.9)	43 (97.7)	12 (100.0)	33 (94.3)	21 (95.5)	58 (95.1)	7 (100.0)	2 (100.0)	21 (100.0)	33 (100.0)	20 (100.0)	10 (90.9)	25 (100.0)	30 (93.8)
Only GBV specialists should work to mitigate risks of GBV	12 (7.1)	4 (4.5)	3 (6.8)	2 (16.7)	3 (8.6)	3 (13.6)	1 (1.6)	1 (14.3)	0 (0.0)	3 (14.3)	1 (3.0)	0 (0.0)	1 (9.1)	1 (4.0)	3 (9.4)
It is not within my scope of work to mitigate risks of GBV	22 (12.9)	9 (10.1)	6 (13.6)	4 (33.3)	10 (28.6)	3 (13.6)	7 (11.5)	1 (14.3)	0 (0.0)	6 (28.6)	3 (9.1)	2 (10.0)	3 (27.3)	3 (12.0)	5 (15.6)
Sector-specific work is a priority over addressing GBV	45 (26.5)	19 (21.4)	13 (29.6)	5 (41.7)	9 (25.7)	9 (40.9)	18 (29.5)	2 (28.6)	1 (50.0)	8 (38.1)	8 (24.4)	7 (35.0)	6 (54.6)	9 (36.0)	9 (28.1)
I would like to work to mitigate risks of GBV, but I do not have the support of my supervisor(s)/senior management to do so	34 (20.0)	13 (14.6)	10 (22.7)	3 (25.0)	8 (22.9)	8 (36.4)	11 (18.0)	2 (28.6)	2 (100.0)	6 (28.6)	8 (24.4)	2 (10.0)	1 (9.1)	6 (24.0)	8 (25.0)
I would like to work to mitigate risks of GBV, but there are limited financial resources	101 (59.4)	59 (66.3)	33 (75.0)	7 (58.3)	23 (65.7)	14 (63.6)	41 (67.2)	5 (71.4)	2 (100.0)	15 (71.4)	24 (72.7)	9 (45.0)	7 (63.6)	16 (64.0)	17 (53.1)
I would like to work to mitigate risks of GBV, but I do not have the time	23 (13.5)	13 (13.6)	8 (18.2)	1 (8.3)	6 (17.1)	1 (4.6)	9 (14.8)	2 (28.6)	0 (0.0)	2 (9.5)	3 (9.1)	1 (5.0)	2 (18.2)	3 (12.0)	5 (15.6)
I would like to work to mitigate risks of GBV, but I do not have the knowledge or skills	48 (28.2)	13 (14.6)	12 (27.3)	2 (16.7)	14 (40.0)	8 (36.4)	17 (27.9)	4 (57.1)	0 (0.0)	5 (23.8)	7 (21.2)	6 (30.0)	4 (36.4)	7 (28.0)	13 (40.6)
**GBV RM knowledge—those with little to knowledge**															
Global guidance on GBV RM in humanitarian programming	41 (24.1)	14 (15.7)	10 (22.7)	2 (16.7)	9 (25.7)	4 (18.2)	15 (24.6)	2 (28.6)	0 (0.0)	6 (28.6)	9 (27.3)	4 (20.0)	2 (18.2)	5 (20.0)	6 (18.8)
How to respond if a survivor discloses an experience of GBV and asks for your help	19 (11.2)	3 (3.4)	3 (6.8)	2 (16.7)	4 (11.4)	4 (18.2)	4 (6.6)	2 (28.6)	0 (0.0)	5 (23.8)	5 (15.2)	1 (5.0)	2 (18.2)	4 (16.0)	5 (15.6)
Measuring GBV RM outcomes in your sector-specific humanitarian programming	43 (25.3)	12 (13.5)	9 (20.5)	4 (33.3)	11 (31.4)	6 (27.3)	9 (14.8)	3 (42.9)	0 (0.0)	8 (38.1)	8 (24.2)	3 (15.0)	5 (45.5)	8 (32.0)	7 (21.9)
Asking about safety perceptions of women and girls in your sector-specific humanitarian programming	33 (19.4)	8 (9.0)	9 (20.5)	2 (16.7)	7 (20.0)	4 (18.2)	13 (21.3)	2 (28.6)	0 (0.0)	7 (33.3)	8 (24.2)	5 (25.0)	2 (18.2)	7 (28.0)	4 (12.5)
**GBV RM Experience & Practices**															
Have implemented GBV RM activities at least once in a humanitarian emergency	112 (65.9)	70 (78.7)	30 (68.2)	10 (83.3)	17 (48.6)	13 (59.1)	37 (60.7)	2 (28.6)	1 (50.0)	15 (71.4)	21 (63.6)	14 (70.0)	8 (72.7)	17 (68.0)	19 (59.4)
Day-to-day work never or rarely involves GBV RM efforts	46 (27.1)	11 (12.4)	10 (22.7)	2 (16.7)	15 (42.9)	6 (27.3)	17 (27.9)	4 (57.1)	1 (50.0)	8 (38.1)	8 (24.2)	6 (30.0)	3 (27.3)	6 (24.0)	10 (31.3)
Is a GBV specialist	74 (43.5)	53 (59.6)	18 (40.9)	4 (33.3)	12 (34.3)	8 (36.4)	23 (37.7)	3 (42.9)	2 (0.0)	5 (23.8)	10 (30.3)	5 (25.0)	3 (27.3)	5 (20.0)	10 (31.3)
If not, never or rarely engages with GBV specialists	50 (52.1)	11 (30.6)	15 (57.7)	4 (50.0)	15 (65.2)	6 (42.9)	21 (55.3)	4 (100.0)	-	8 (50.0)	14 (60.9)	9 (60.0)	5 (62.5)	7 (35.0)	14 (63.6)

GBV RM: GBV risk mitigation.
